# Marine Reserves Enhance the Recovery of Corals on Caribbean Reefs

**DOI:** 10.1371/journal.pone.0008657

**Published:** 2010-01-11

**Authors:** Peter J. Mumby, Alastair R. Harborne

**Affiliations:** Marine Spatial Ecology Lab, School of BioSciences, Hatherly Laboratory, University of Exeter, Exeter, United Kingdom; Smithsonian's National Zoological Park, United States of America

## Abstract

The fisheries and biodiversity benefits of marine reserves are widely recognised but there is mounting interest in exploiting the importance of herbivorous fishes as a tool to help ecosystems recover from climate change impacts. This approach might be particularly suitable for coral reefs, which are acutely threatened by climate change, yet the trophic cascades generated by reserves are strong enough that they might theoretically enhance the rate of coral recovery after disturbance. However, evidence for reserves facilitating coral recovery has been lacking. Here we investigate whether reductions in macroalgal cover, caused by recovery of herbivorous parrotfishes within a reserve, have resulted in a faster rate of coral recovery than in areas subject to fishing. Surveys of ten sites inside and outside a Bahamian marine reserve over a 2.5-year period demonstrated that increases in coral cover, including adjustments for the initial size-distribution of corals, were significantly higher at reserve sites than those in non-reserve sites. Furthermore, macroalgal cover was significantly negatively correlated with the change in total coral cover over time. Recovery rates of individual species were generally consistent with small-scale manipulations on coral-macroalgal interactions, but also revealed differences that demonstrate the difficulties of translating experiments across spatial scales. Size-frequency data indicated that species which were particularly affected by high abundances of macroalgae outside the reserve had a population bottleneck restricting the supply of smaller corals to larger size classes. Importantly, because coral cover increased from a heavily degraded state, and recovery from such states has not previously been described, similar or better outcomes should be expected for many reefs in the region. Reducing herbivore exploitation as part of an ecosystem-based management strategy for coral reefs appears to be justified.

## Introduction

With increasing rates of global change, the need to conserve key ecosystem services, largely through conservation measures, is greater than ever [1]. In many cases, the implementation of conservation measures for dealing with global change involves a short-term economic cost to local stakeholders and adoption of conservation practices is most likely to be successful when the impacts of the conservation tool are demonstrably beneficial [2]. Frequently, however, the efficacy of conservation tools, such as reserves, is incompletely understood or controversial. This problem is amply demonstrated on coral reefs, where no-take marine reserves are the most widely-used conservation tool [3], [4]. While the efficacy of reserves in promoting biodiversity and fish biomass by reducing local-scale stressors has been widely documented [5]–[7], there is an increasing desire to establish whether reserves can also build coral resilience and offset the effects of global climate change that elevate coral mortality and constrain coral calcification [8], [9].

In Caribbean systems, protecting large herbivorous fishes from fishing can generate a trophic cascade that reduces the cover of macroalgae [10], which is a major competitor of corals [11], [12]. In principle, such a shift in benthic community structure should facilitate the recovery of coral populations after bleaching events, or indeed other disturbance events such as hurricanes, that cause sudden and extensive coral mortality [13], [14]. Thus, reserves in Caribbean systems have the potential to increase the resilience of coral to climate change [15], and thereby enhance the long-term services provided by these systems, such as coastal defence, tourism, and fisheries [16]. However, reserves have not yet been demonstrated to enhance coral recovery [17].

There are several explanations for the lack of data demonstrating the effects of entire reserves on coral recovery. Small-scale experimental manipulations have demonstrated that drastic reductions in fish grazing can cause harmful macroalgal blooms and reduce recovery of corals following bleaching-induced mortality [18]. While these results imply that the conservation of herbivores inside marine reserves should benefit coral recovery, extrapolating small-scale experiments to the spatial scale at which management occurs can be problematic. For example, experimental manipulations that use cages to exclude most fish do not necessarily represent conservation interventions, where relatively modest changes in fish communities are expected. Demonstrating larger-scale, *in situ* reserve impacts are also challenging because disturbances and variations in initial benthic community compositions complicate the attribution of cause and effect on individual reef trajectories.

We studied coral population dynamics at 10 sites throughout the Exuma Cays (Bahamas; Supporting Information Figure S1) over a 2.5 year period (2004–2007) in order to contrast the trajectories of coral populations both inside and outside reserves. Four sites were located in the Exuma Cays Land and Sea Park (ECLSP), a large reserve (456 km^2^) that was designated in 1958 and enforced by wardens since 1986. Importantly, because the reserve location was not biased by the quality of reefs contained [19] and natural processes of larval supply do not appear to differ significantly between reserve and neighbouring reefs [20], the reserve serves as a large-scale experimental study of fishing impacts on ecosystem processes [10], [20]–[22]. Previous studies in the ECLSP have shown that a doubling of parrotfish biomass in the reserve has reduced the cover of their macroalgal prey fourfold and that this reduction in macroalgae has led to an increase in the density of juvenile corals [10], [20]. Indeed, the cover of macroalgae is strongly, linearly, and negatively related to the extent of parrotfish grazing across reefs in this region of the Bahamas (Supporting Information Figure S2, *r^2^* = 0.68, P = 0.004). A 2.5 year period was considered long enough to detect changes in coral cover yet short enough that differences in the trajectories among sites were not heavily influenced by multiple stochastic disturbance events.

## Results and Discussion

**Figure 1:**
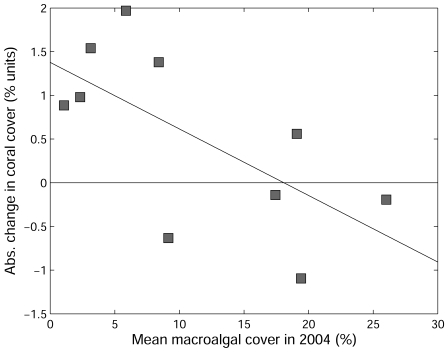
Effect of macroalgal cover on the absolute change in total coral cover at survey sites. Changes were between 2004 and 2007 for 10 sites in the Exuma Cays, Bahamas. The four sites in the Exuma Cays Land and Sea Park had the lowest macroalgal cover.

Because the Bahamas was severely disturbed by the 1998 coral bleaching event [23], and later by hurricane Frances in the summer of 2004, coral cover was low at the beginning of the study, averaging only 7% at reserve and non-reserve sites (Supporting Information Table S1). The proportional increase in coral cover after 2.5 years was fairly high at reserve sites (mean of 19% per site) and significantly greater (one-tailed t-test P = 0.004) than that in non-reserve sites which, on average, exhibited no net recovery. A mechanistic insight into the change in coral cover was sought using regression onto the cover of macroalgae at the start of the study (Figure 1). Macroalgal cover explained 43% of the variance in the change in total coral cover over time (P = 0.041). Coral cover increased at sites with relatively low macroalgal cover but declined at sites with higher cover. The change in cover was mostly driven by two diminutive brooding species of coral (*Porites astreoides* and *Agaricia agaricites*) and one framework-building species, *Montastraea annularis*. In each of these species, the overall pattern of recovery contrasted across park boundaries, showing net recovery (increase in percentage cover) inside the park but net mortality outside (one-tailed Mann-Whitney U-test, P<0.05 for the brooders though only marginally significant for *M. annularis* at P = 0.068). The change in cover of *Agaricia* and *Porites* was moderately-strongly and negatively related to macroalgal cover (*r^2^* = 0.46, P<0.03 in both species) but a relationship with macroalgal cover was not evident for the trajectory of *M. annularis*.

Although trajectories of coral cover were positive inside reserves and generally negative outside reserves, our results were potentially biased by differences in the initial size-distribution of corals which varied significantly among sites in several species, including *A. agaricites* and *M. annularis* (Kolmogorov-Smirnoff test, P<0.05). Bias is possible because coral populations of equivalent cover but different size distributions have strikingly different scope for recovery. Imagine a series of reefs, each with identical coral cover, but some comprise a few large corals whereas others comprise many small corals. As encrusting corals grow in a linear, radial fashion [24] the final coral cover after, say, 1 year of growth is substantially greater in the community dominated by many small colonies (e.g., if the initial cover comprised 20 small colonies then the absolute increase of cover would be six times greater than a community of identical initial cover that comprised a single large colony). To address this problem we developed an abstract alternative measure using Monte Carlo simulation that took the initial size distribution of each species at each site and found the radial growth rate that most closely accounted for the difference in total cover between sampling intervals. The process was repeated at each site giving an overall ‘size-adjusted rate of change of cover’ (SARCC) for each coral species based on the size distribution and observed change in coral cover at that site. Although SARCC is calculated as a linear extension rate of coral it does not directly represent a radial growth rate because it is a population-level property that subsumes coral colonisation, growth, shrinkage and mortality. However, basing its calculation on the radial growth of individual corals has the desirable property of explicitly incorporating the initial size distribution of corals. It is not intended to offer any demographic insight other than if the value is positive then recruitment and growth outweigh mortality and *vice versa* (the properties of SARCC are discussed further in the Materials and Methods).

**Figure 2 pone-0008657-g002:**
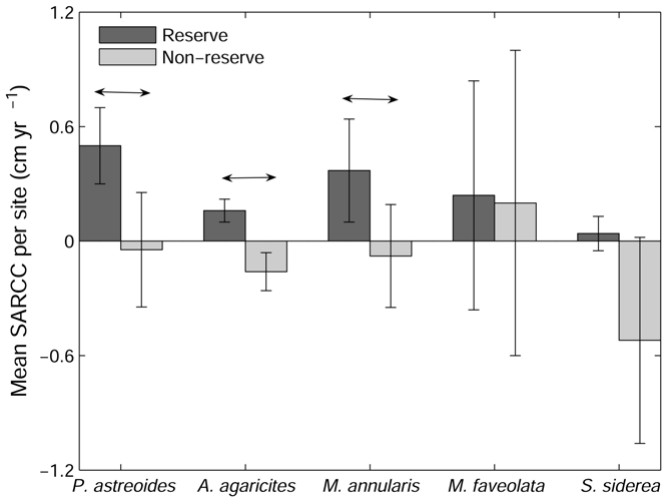
Size-adjusted rate of change of cover (SARCC) of dominant coral species at survey sites. Sites were inside and outside of the Exuma Cays Land and Sea Park, Bahamas. Error bars denote s.e.m. Horizontal arrow denotes significant differences (one-tailed t-test P<0.05).

**Figure 3:**
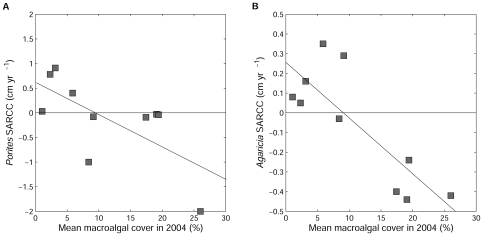
Effect of macroalgal cover on the size-adjusted rate of change of cover (SARCC). Panels show relationships for SARCC of *Porites astreoides* (A) and *Agaricia agaricites* (B) between 2004 and 2007 for 10 sites in the Exumas Cays, Bahamas. The four reserve sites had the lowest macroalgal cover.

Repeating our analyses with SARCC instead of absolute or proportional change in coral cover did not alter our conclusions (Figures 2 and 3). However, the difference in SARCC between reserve and non-reserve sites for *M. annularis* moved from marginal (P = 0.068) to clear significance (P = 0.018), and macroalgal cover explained a greater proportion of the variance in SARCC of *A. agaricites* (*r^2^* = 0.59, P = 0.009).

We also subjected our analysis to one further refinement in light of the coral bleaching event of 2005 [14]. Although coral bleaching was not severe in the Bahamas [14] (also confirmed by *in situ* observations at the study sites, Mumby pers. obs.), we calculated the accumulated thermal stress in 2005 above that of the climatological maximum monthly mean [25]. We then asked whether differences in thermal stress constituted a plausible alternative explanation of our results to that of macroalgal cover. Adding accumulated thermal stress in a linear model against either absolute change in coral cover, proportional change in cover or SARCC did not result in a significant coefficient. In fact, the most severe thermal stress was encountered at one site in the ECLSP and no significant differences were found between the stress experienced at reserve and non-reserve sites.

**Figure 4:**
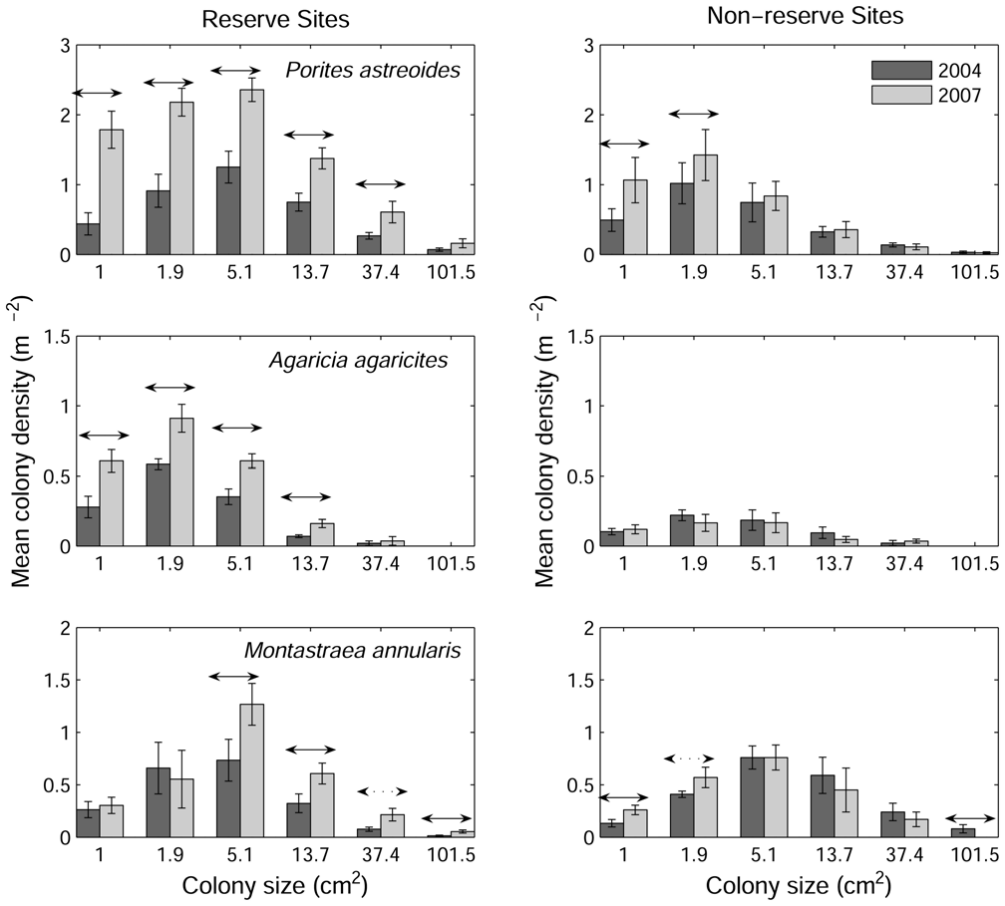
Size distributions of coral density in three coral species. Size distributions highlight changes over time (2004 to 2007) and differences between reserve and non-reserve sites. Error bars denote s.e.m. from site-averaged data. Horizontal arrows denote significant differences (one-tailed t-test, solid arrows for P<0.05, dashed arrows for 0.05<P<0.1).

Some of the most abundant macroalgae on Caribbean reefs, such as *Lobophora variegata* and *Dictyota pulchella*, compete with corals through a variety of mechanisms including direct overgrowth [12], [26], pre-emption of settlement space and reduced colony growth rate [27]. Our data do not allow us to disentangle the detailed way in which macroalgae influence coral recruitment, growth and mortality because there are many ways in which demographic processes can generate the observed size distributions [28] and additional data on demographic rates would be required. However, our results do provide some insight into macroalgal impacts at population scales. Comparing the size structure of coral populations from 2004 to 2007 reveals a striking difference between reserve and non-reserve sites (Figure 4), that complements the analyses of coral cover trajectories (Figures 1–3). Coral populations exhibited a healthy demographic flux inside reserves with colonies growing from smaller size classes to larger classes (Figure 4). In the case of *Porites* and *Agaricia*, the increase in smaller size classes in 2007 was partly due to continued recruitment between census dates but successful somatic growth of established colonies also took place because new recruits could not have grown large enough to reach the fifth and fourth size classes (for *Porites* and *Agaricia* respectively) in the time elapsed between census dates. In contrast, coral populations outside the reserve lacked the demographic succession among size classes that was observed inside the reserve, implying that populations were, on average, not recovering (Figure 4). Relatively little recruitment was observed in *Porites* outside reserves and the density of colonies in larger size classes either remained stable or declined over time (Figure 4), strongly implying that a macroalgal-induced population bottleneck restricts the supply of smaller corals to larger size classes. The degree to which this bottleneck is caused by macroalgal impacts on colony somatic growth or mortality cannot be determined definitively from our data though the identification of a population bottleneck is consistent with small-scale field experiments [27] and predictions from ecological models [29]. The bottleneck appeared to be even more extreme in *Agaricia* where there was no sign of net recruitment or growth outside the reserve, a pattern in stark contrast to that observed within the reserve (Figure 4).

The mechanisms driving change in the recovery of *M. annularis* are more difficult to identify. Recruitment occurs rarely in this species and the increased densities found in the smaller size classes outside the reserve were almost entirely attributable to fission of established colonies rather than recruitment. Colony somatic growth appears to have occurred across a range of size classes inside the reserve but not so outside its boundaries; indeed a significant decline occurred in the largest size class (Figure 4). The role of macroalgae in arresting recovery outside the reserve is unclear given the lack of a simple linear relationship. Contact with macroalgae certainly has energetic costs for *M. annularis* [30] but if algae are a cause of diminished recovery, the relationship may either be complex or simply difficult to measure, possibly because of the high susceptibility of *M. annularis* to disease [31] which may obscure the effects of processes like algal competition.

Most studies of macroalgal impacts on coral have taken place in small experimental plots and our results provide new insight into the scalability of such studies from individual to population scales. Experimental manipulations have found that *A. agaricites* is highly susceptible to macroalgal overgrowth [32], and our study suggests that this conclusion is borne out at ecosystem scales. Experimental studies of macroalgal impacts on *P. astreoides* led us to expect a weaker impact than that found for *A. agaricites* because *P. astreoides* has been found to be relatively resistant to *Lobophora* encroachment [32] and contact with *Dictyota* has reduced coral growth rate but not led to mortality [26]. Again, this *a priori* expectation was generally supported because, despite some inter-site variation (Figure 3), mean *Porites* SARCC outside the reserve appeared to be in near-stasis (Figure 2) whereas *Agaricia* exhibited a sharp contraction (negative SARCC; Figure 2). Further, comparing the relative magnitudes of contraction outside the reserve and expansion inside the reserve (Figure 2) shows that the proportional level of contraction is tenfold weaker in *Porites* than *Agaricia* (contraction/expansion 0.05/0.5 vs. 0.16/0.16 respectively, Figure 2).

The response of large spawning corals to a gradient of macroalgal cover exhibited a variable fit to experimental predictions. Previous studies have found *Siderastrea siderea* to be unaffected by *Dictyota* contact [26] which is consistent with the absence of a significant effect in our study (Figure 2). In contrast, *Montastraea faveolata* has been found to be highly susceptible to algal overgrowth [26] whereas we found no effect (Figure 2) despite *Lobophora* and *Dictyota* being common in our study area [22]. Our finding of mixed levels of scalability from experimental outcomes to ecosystem-level effects in the Bahamas in no way implies criticism of the original experiments. However, it does reinforce the need to repeat experiments in different biophysical environments and test their scalability under a variety of conditions; a process that is rarely attempted.

Marine reserves cannot protect corals from direct climate-induced disturbance [17], but they can increase the post-disturbance recovery rate of some corals providing that macroalgae have been depleted by more abundant communities of grazers that benefit from reduced fishing pressure. Such trophic cascades are most likely in the Caribbean because of the depauperate herbivore community and increased functional importance of parrotfishes following a disease-induced mortality event that significantly reduced densities of a major herbivore taxon, the urchin *Diadema antillarum* [33]. The only other study that has attempted to quantify trajectories of coral populations inside and outside of reserves was conducted in the Indian Ocean, and found insignificant differences in coral cover growth rates [34]. The higher diversity of herbivores compared to Caribbean reefs, and therefore smaller differences in trophic cascades between fished and unfished reefs, is likely to have been an important factor limiting the effect of the reserves on coral recovery rates.

While the absolute rate of coral recovery in the ECLSP was low, it must be borne in mind that these reefs had little coral to start with and that recovery trajectories would normally accelerate as corals recover [35]. The degree to which reserve-driven rates of recovery will buffer the anticipated rise in rate of coral bleaching, disease, and severe hurricanes is currently unclear and will undoubtedly vary regionally [29]. Indeed, coral cover does not appear to be increasing in some Caribbean reserves [36] and the causation might include overwhelming coral mortality, a lack of reserve impacts on fish, or a lack of herbivore impacts on the benthos if other processes, such as nutrification or sedimentation, were to dominate the response of algae. Nonetheless, it is perhaps significant that the first documentation of net recovery from a heavily-depleted Caribbean coral community (<10% cover) stems from one of the region's most successful marine reserves. The need to take local action to reduce anthropogenic stress on reefs is both warranted and urgent.

## Materials and Methods

### Study Sites

Surveys were conducted at the same sites in and around the Exuma Cays Land and Sea Park (ECLSP) in October 2004 and May 2007. The location of the ECLSP was the result of a general desire for conservation in The Bahamas, and the availability of Crown Land in the Exuma Cays relatively close to the tourism centre of Nassau [19]. There is no evidence of the reserve containing especially healthy or diverse reefs before its establishment, and the entire Exuma Cays remain an area of relatively low population density and limited land-based pollution. A ban on fishing has been enforced by warden patrols since 1986. Poaching inside the ECLSP has been assessed as low [3]. Of the large commercial fishing vessels registered as fish traps in the Bahamas, 40% have sufficient size (>10 m) and are in close enough proximity (Nassau to Exuma Cays) to fish around the reserve [10]. In addition, 30 fish traps are deployed locally to the south of the reserve. Although such traps are used to target grouper species, they result in bycatch of parrotfishes [37]. The reduction in fishing inside the reserve has led to higher densities of fish and invertebrates than found outside the reserve [10], [21], [22]. All surveys were conducted within the forereef habitat ‘*Montastraea* reef’ (coral-rich areas visually dominated by *Montastraea* spp.), which has the greatest diversity and density of all fish and invertebrates in the Bahamas [38]. This habitat was sampled at four sites (≈150 m×≈150 m) near the centre of the ECLSP, three sites between 5.8 and 18.1 km north of the park, and three sites around Lee Stocking Island ≈70 km south of the park (Figure S1). The same sites were used during both survey periods and identified by GPS co-ordinates, and are on a continuous stretch of forereef. The depth at each site varied from 8–17 m.

### Benthic Surveys and Derivation of Coral Cover and Size-Frequency Data

At each survey site between 28 and 99 (mean 42.9) randomly-placed 1 m^2^ quadrats were used to quantify coral and macroalgal cover. Content of quadrats was filmed in 20 cm swathes, using a high-resolution digital video camera. After the swathes were filmed, a second pass of the camera was made close to the substrate surface to reveal cryptic substrata on the sides of structure or under macroalgae. Coral and macroalgal covers were assessed at each site using the Vidana software [freely available from www.ex.ac.uk/msel]. Individual corals were identified to species level and their cover (size) was measured using Vidana within five randomly sub-sampled 0.04 m^2^ quadrats in every 1 m^2^ quadrat. The smallest corals censused by using this technique had a diameter of ≈1 cm. Corals that extended beyond quadrat boundaries were noted and removed from analyses of size distributions. More than 9,000 individual corals (>6000 for 2004, >3000 for 2007) were sampled. Although it was most appropriate to regress coral recovery onto macroalgal cover at the start of the study (i.e., 2004 which represented the level of algal cover from which corals had to recover), we also verified that the pattern of macroalgal cover persisted throughout the study. Thus, the regression of change in coral cover against macroalgal cover in 2007 was also significant, linear and negative (*r^2^* = 0.63, P = 0.006) as it was using 2004 data.

### Calculation and Properties of SARCC for Individual Coral Species

A Monte Carlo method was used to determine SARCC. For each species and site the algorithm generated a probability distribution of finding corals in each of 10 size classes in 2004. The ten equally spaced categories of size were allowed to vary among species as appropriate. The size distribution was converted into a probability distribution by calculating the number of colonies in size class *i* as a proportion of the total number of colonies, *n*. The probability distribution was then used to generate a virtual sample of corals with the same areal cover as that species at that site. Each colony within the virtual sample was then allowed to grow for 30 months, using a fixed putative SARCC varying from −3 (contraction) to +3 cm yr^−1^ (expansion), in 0.05 increments. SARCC was applied as a linear radial extension rate, in a manner consistent with growth studies in corals [39]. For each possible SARCC, the total predicted cover of the species at the end of 30 months was compared to that observed in our 2007 surveys. The SARCC that resulted in the closest match between predicted and observed cover was recorded. As an example of the algorithm, if an annual growth rate of 8 mm yr^−1^ was applied to the individual colonies of *P. astreoides* observed in 2004 at site 2 of the ECLSP, then the predicted total cover of these colonies when we returned to resurvey in 2007 would match that observed. In this case, the rate of 8 mm yr^−1^ was found heuristically by trying many possible growth rates. The entire process was then repeated for a minimum of 100 virtual coral samples. Finally, the mean of the most accurate results for SARCC was calculated. The disparity in predicted and observed coral covers associated with the selected SARCC were within 0.05 of 1%.

SARCC is an abstract concept that reflects the net expansion or contraction of the entire coral population, weighted appropriately for the initial size distribution of corals. Here we summarise its desirable properties and limitations. SARCC is not a tool for demographic analysis because it subsumes processes of recruitment, growth and mortality. However, it is important to appreciate the impact of different demographic processes on SARCC.

Desirable properties of SARCC for comparisons among sites:

1. If all sites have *identical rates of recruitment, growth, and mortality and similar initial size distribution* (but perhaps different initial abundance) then they will have identical SARCC.
2. If all sites have identical demographic rates but differ in their *initial size distribution*, then SARCC is a direct measure of potential recovery rate and differs among sites appropriately
3. If all sites are identical with the exception of having *different levels of recruitment* then SARCC will be positively correlated with recruitment rate. This is appropriate because SARCC will have to ‘grow faster’ to account for the higher-than-expected coral cover at sites with greater rates of recruitment. After all, coral cover will have increased at a faster rate as these sites.
4. If all sites are identical with the exception of having *different levels of mortality rate* then SARCC will be negatively correlated to the pattern of mortality rate. This is appropriate because SARCC will have to select low radial growth rates (possibly negative rates) in order to match the lower-than-expected coral cover. Negative values of SARCC are possible and imply a contraction of coral colonies.
5. If all sites are identical with the exception of having *different levels of coral growth rate* then SARCC will reflect this directly and SARCC can be interpreted as a rate of colony somatic growth under these circumstances.

Limitations of SARCC:

1. SARCC should not be interpreted as a measure of somatic coral colony growth unless other demographic processes are identical among populations (which is unlikely).
2. Alone, SARCC does not quantify the relative importance of demographic processes other than if the SARCC is positive then recruitment and growth outweighs mortality and colony shrinkage (and the opposite applies if SARCC is negative).
3. In principle, two sites could have the same SARCC but very different underlying demographic processes; however, the absolute change in coral cover would also be identical so this simply underscores the inability of SARCC to reveal the *relative* rates of recruitment, growth and mortality.

### Size-Distribution of Corals

To examine changes in the size distribution of coral colonies, corals were binned into six categories following a log scale (<1, 1.01–2.72, 2.73–7.39, 7.40–20.09, 20.10–54.60, 54.61–148.41 cm^2^). Note that larger colonies existed in *Montastraea annularis* in 2004 but their frequency was so low that they were omitted from plots. To determine whether an increase in the density of colonies within a size category could be due to recruitment alone, we determined the maximum expected size of corals if they had recruited at some point after the first survey in 2004. Growth rates were extracted from van Moorsel's detailed observations of growth of *Porites* and *Agaricia* in this size range (1.9 mm mo.^−1^ and from 0.05 mm mo.^−1^ to 1 mm mo.^−1^ respectively) [39]. For *Porites astreoides*, the mean growth rate implied that no coral could have reached size class 6 (54.60–148.41) and only a small fraction could have reached size class 5 (20.10–54.60) as this would require 27 months growth. Even allowing for the fastest published growth rate for *Agaricia agaricites*, corals would not have reached size class 4 if recruiting between October 2004 and May 2007.

## Supporting Information

Figure S1 Location of survey sites in and around the Exuma Cays Land and Sea Park.
(0.68 MB TIF)

Figure S2 Relationship between parrotfish grazing intensity and macroalgal cover at 10 sites in the Exumas.
(0.58 MB TIF)

Table S1 Absolute and proportional change in coral cover, plus macroalgal cover, at each site surveyed.
(0.04 MB DOC)
