# Supplementary figures and images for: Marine Reserves Enhance the Recovery of Corals on Caribbean Reefs
Source: PLoS One. 2010 Jan 11;5(1):e8657. doi: 10.1371/journal.pone.0008657 (PMC2799675; doi:10.1371/journal.pone.0008657)

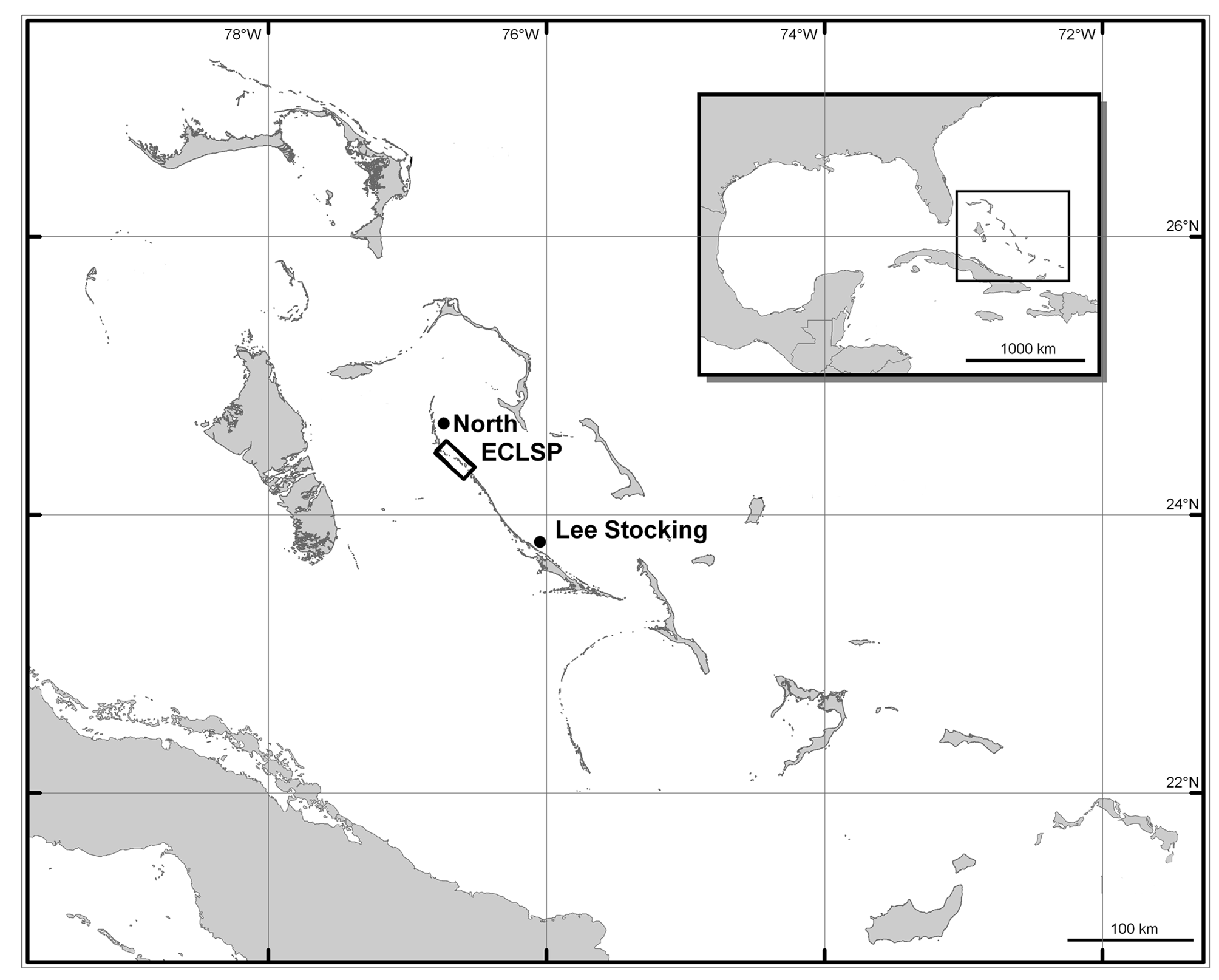

Supplement: Figure S1 — Location of survey sites in and around the Exuma Cays Land and Sea Park. (0.68 MB TIF) [file pone.0008657.s001.tif]

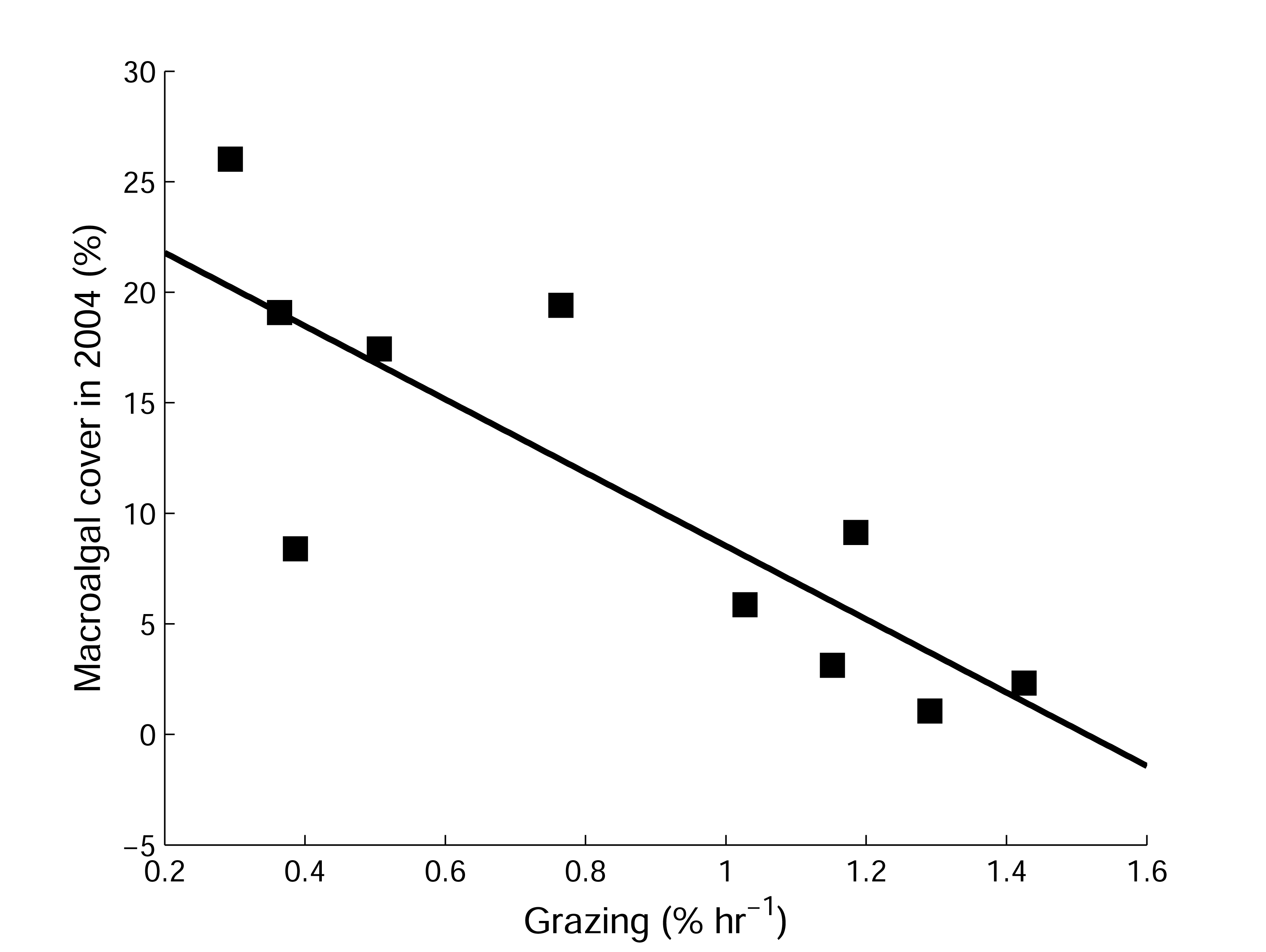

Supplement: Figure S2 — Relationship between parrotfish grazing intensity and macroalgal cover at 10 sites in the Exumas. (0.58 MB TIF) [file pone.0008657.s003.tif]
