# Supplementary material for: Marine Reserves Enhance the Recovery of Corals on Caribbean Reefs
Source: PLoS One. 2010 Jan 11;5(1):e8657. doi: 10.1371/journal.pone.0008657 (PMC2799675; doi:10.1371/journal.pone.0008657)
Supplement: Table S1 — Absolute and proportional change in coral cover, plus macroalgal cover, at each site surveyed. (0.04 MB DOC) [file pone.0008657.s002.doc]

|  |  |  |  |  |  |  |  |  |
| --- | --- | --- | --- | --- | --- | --- | --- | --- |
| **Location** | **Site** | **Number of quadrats in 2004** | **2004 coral cover (%) (SE)** | **Number of quadrats in 2007** | **2007 coral cover (%) (SE)** | **Absolute change in coral cover 2004-2007** | **Proportional change in coral cover 2004-2007** | **2004 macroalgal cover (%)**  **(SE)** |
| North of Park | 1 | 37 | 5.0 (0.4) | 28 | 4.4 (0.8) | -0.63 | -0.13 | 9.1 (1.4) |
|  | 2 | 40 | 2.6 (0.5) | 62 | 2.4 (0.4) | -0.14 | -0.05 | 17.4 (3.7) |
|  | 3 | 41 | 3.4 (0.8) | 40 | 3.9 (0.9) | +0.56 | +0.16 | 19.1 (3.5) |
| Inside Park | 1 | 39 | 9.7 (1.1) | 99 | 11.6 (0.7) | +1.97 | +0.20 | 5.9 (1.3) |
|  | 2 | 40 | 8.6 (1.2) | 40 | 9.5 (1.1) | +0.98 | +0.11 | 2.3 (0.4) |
|  | 3 | 40 | 5.9 (0.9) | 40 | 7.5 (1.3) | +1.54 | +0.26 | 3.1 (0.4) |
|  | 4 | 37 | 4.4 (0.4) | 41 | 5.3 (0.9) | +0.89 | +0.20 | 1.0 (0.2) |
| South of Park | 1 | 41 | 15.5 (2.0) | 39 | 16.8 (2.6) | +1.38 | +0.09 | 8.4 (1.0) |
|  | 2 | 40 | 14.7 (1.6) | 40 | 13.6 (1.7) | -1.10 | -0.07 | 19.4 (1.7) |
|  | 3 | 43 | 3.4 (0.6) | 31 | 3.3 (1.2) | -0.19 | -0.06 | 26.0 (1.8) |
